# Identification of a 4-fluorobenzyl l-valinate amide benzoxaborole (**AN11736**) as a potential development candidate for the treatment of Animal African Trypanosomiasis (AAT)

**DOI:** 10.1016/j.bmcl.2017.11.028

**Published:** 2018-01-01

**Authors:** Tsutomu Akama, Yong-Kang Zhang, Yvonne R. Freund, Pamela Berry, Joanne Lee, Eric E. Easom, Robert T. Jacobs, Jacob J. Plattner, Michael J. Witty, Rosemary Peter, Tim G. Rowan, Kirsten Gillingwater, Reto Brun, Bakela Nare, Luke Mercer, Musheng Xu, Jiangong Wang, Hao Liang

**Affiliations:** a Anacor Pharmaceuticals, Inc., 1020 E. Meadow Circle, Palo Alto, CA 94303, USA; b Global Alliance for Livestock and Veterinary Medicine, Doherty Building, Pentlands Science Park, Penicuik, Edinburgh EH26 0PZ, UK; c Swiss Tropical and Public Health Institute, Socinstrasse 57, 4051 Basel, Switzerland; d University of Basel, Petersplatz 1, 4003 Basel, Switzerland; e Avista Pharma Solutions, 350 Tricenter Boulevard, Suite C, Durham, NC 27713, USA; f Wuxi AppTec (Tianjin) Co. Ltd., No. 168 NanHai Road, 10th Avenue, TEDA, Tianjin 300457, PR China

**Keywords:** Benzoxaborole, Trypanosomiasis, Cattle, SAR, Lead optimisation, Protozoan

## Abstract

Novel l-valinate amide benzoxaboroles and analogues were designed and synthesized for a structure-activity-relationship (SAR) investigation to optimize the growth inhibitory activity against *Trypanosoma congolense* (*T. congolense*) and *Trypanosoma vivax* (*T. vivax*) parasites. The study identified 4-fluorobenzyl (1-hydroxy-7-methyl-1,3-dihydrobenzo[*c*][1,2]oxaborole-6-carbonyl)-l-valinate (**5**, **AN11736**), which showed IC_50_ values of 0.15 nM against *T. congolense* and 1.3 nM against *T. vivax*, and demonstrated 100% efficacy with a single dose of 10 mg/kg against both *T. congolense* and *T. vivax* in mouse models of infection (IP dosing) and in the target animal, cattle, dosed intramuscularly. **AN11736** has been advanced to early development studies.

## Graphical abstract

**Figure:**
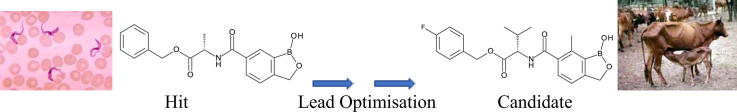

Animal African Trypanosomiasis (AAT) is a fatal, parasitic wasting disease of livestock and wild animals in Sub-Saharan Africa.^1^ It is caused primarily by the two protozoan parasites *Trypanosoma congolense* (*T. congolense*) and *Trypanosoma vivax* (*T. vivax*)*,* which are transmitted by tsetse flies.^1^ AAT is responsible for 3 million cattle deaths annually and costs African livestock farmers approximately US$ 1–5 billion per year.^2^ The current standard-of-care drugs, such as diminazene aceturate, isometamidium and homidium chloride, have been in use for several decades and are often ineffective with drug resistance becoming an increasing concern.^1^ No new trypanocides have been approved for use in cattle for many years. Initial screening of the Anacor Pharmaceuticals library of novel boron-containing compounds identified an active compound (**1**, Fig. 1), which had an IC_50_ = 5 nM against *T. congolense* and 69 nM against *T. vivax* while its enantiomer was much less active. A quick and simple modification on the amino acid side chain with an isopropyl group generated **2** (Fig. 1) with improved *in vitro* potency (IC_50_ = 2 nM against both *T. congolense* and *T. vivax*). This encouraging result prompted us to investigate this chemical series further. We designed and synthesized a series of novel benzoxaboroles (**3**–**71**, Fig. 2, Fig. 3, Fig. 4, Fig. 5, Fig. 6, Fig. 7, Fig. 8) to optimize anti-parasitic activity, physicochemical and *in vitro* ADME properties, and the pharmacokinetic profile. Specifically, these molecules were designed to examine the effects of oxaborole 3-substituent variation (**3** vs **2**, Fig. 1, Fig. 2), oxaborole 7-substituent variation (**4** vs **2**, Fig. 1, Fig. 2; **5** vs **20**–**27**, Fig. 4), substituent changes on the benzyl group (**5**–**19**, Fig. 3), modification of the amino acid (**28**–**32**, Fig. 5), heteroaromatic methyl esters (**33**–**48**, Fig. 6), introduction of water-solubilizing scaffolds to the benzyl group (**49**–**54**, Fig. 7) and aliphatic esters (**55**–**71**, Fig. 8). Herein, we report the synthesis and antiparasitic activity against *T. congolense* and *T. vivax* of these novel compounds.

**Fig. 1:**
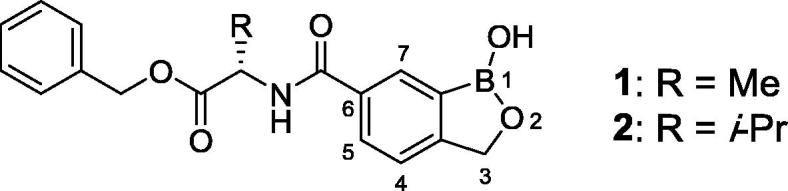
Chemical structures of early hits (**1** and **2**).

**Fig. 2:**
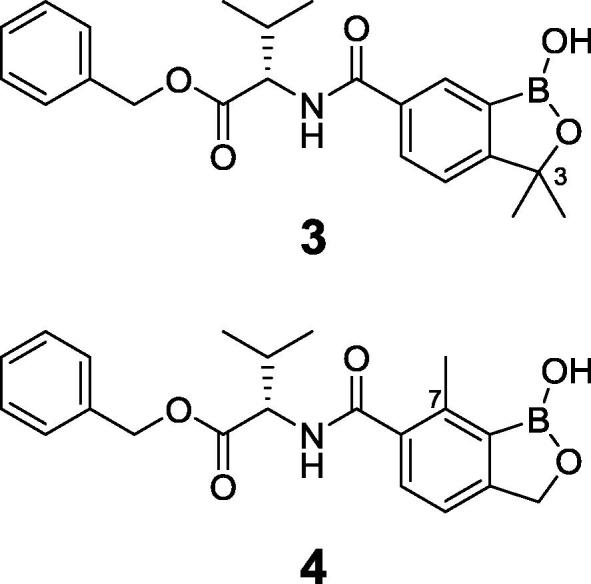
Structures of benzoxaboroles with additional 3,3-Me_2_ (**3**) or 7-Me (**4**) modification as compared to analog **2**.

**Fig. 3:**
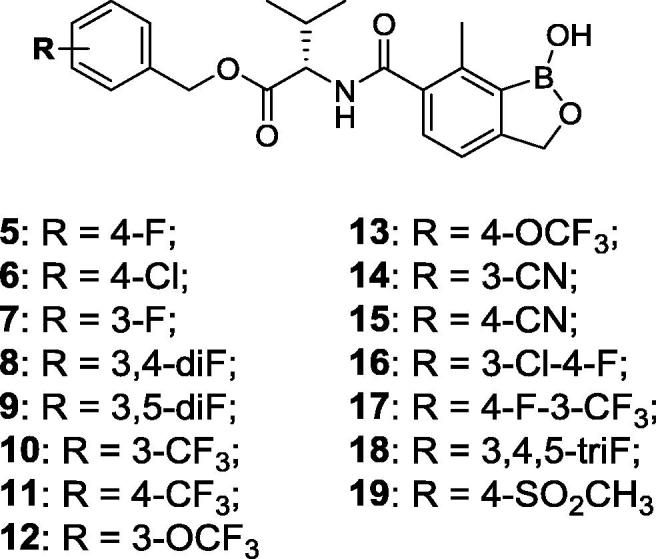
Structures of benzoxaboroles with variation of substituents on the benzyl ring (**5**–**19**) as compared to analog **4**.

**Fig. 4:**
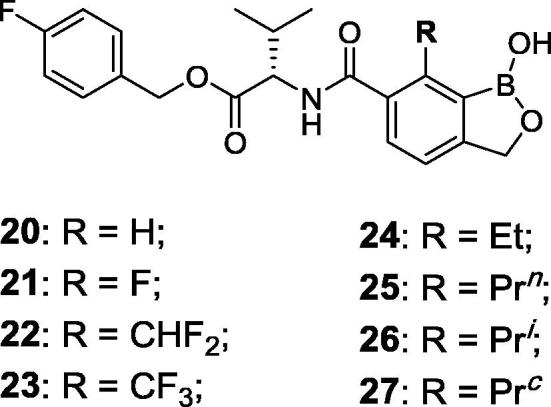
Structures of benzoxaboroles with variation of 7-substituents on the benzene ring (**20**–**27**) as compared to analog **5**.

**Fig. 5:**
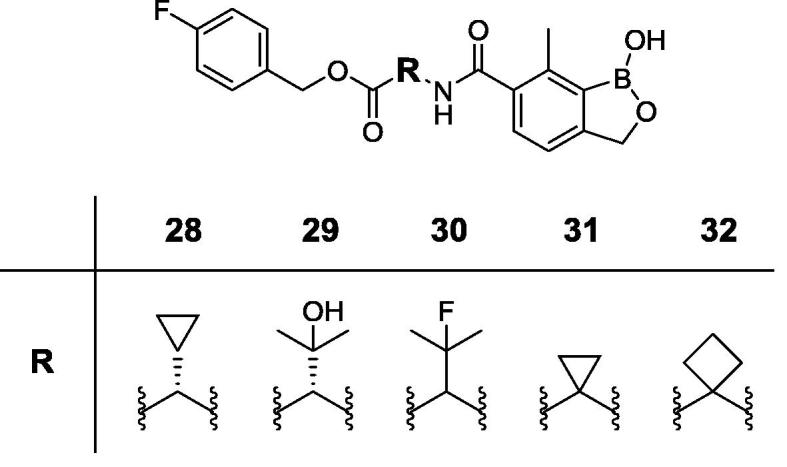
Structures of benzoxaboroles with variation on the amino acid side chain (**28**–**32**) as compared to analog **5**.

**Fig. 6:**
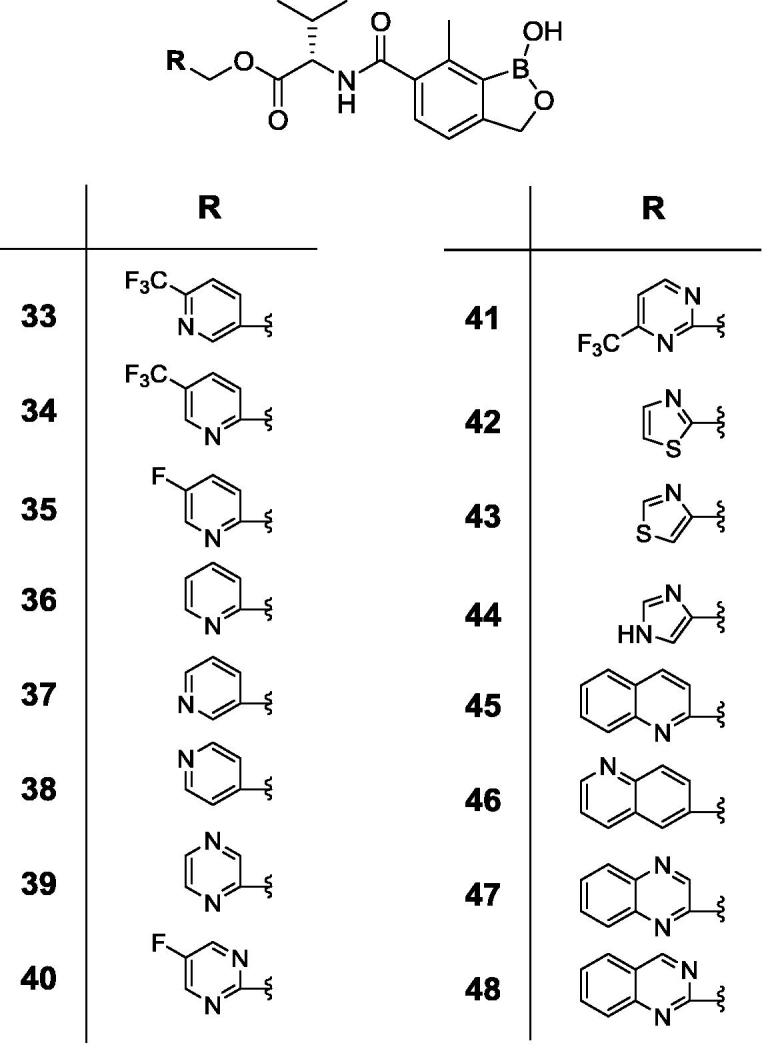
Structures of benzoxaboroles with variation of the left side arylmethyl groups (**33**–**48**) as compared to analog **5**.

**Fig. 7:**
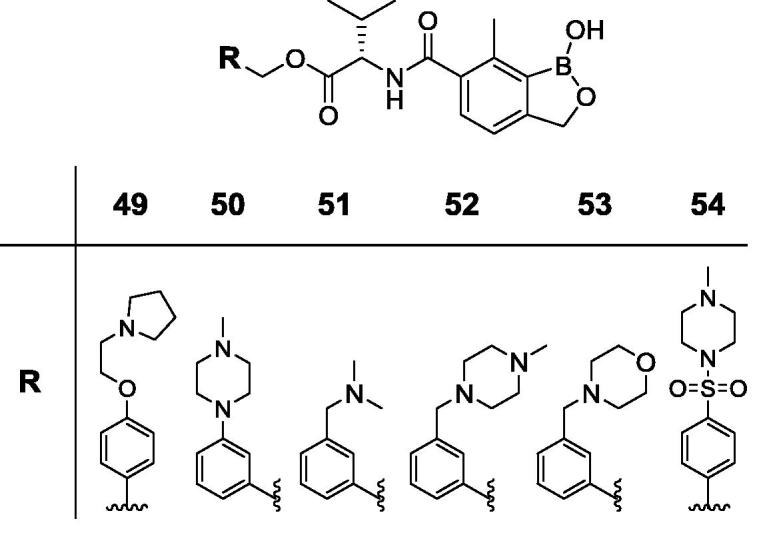
Structures of benzoxaboroles with water-solubilizing scaffolds on the benzyl ring (**49**–**54**) as compared to analog **5**.

**Fig. 8:**
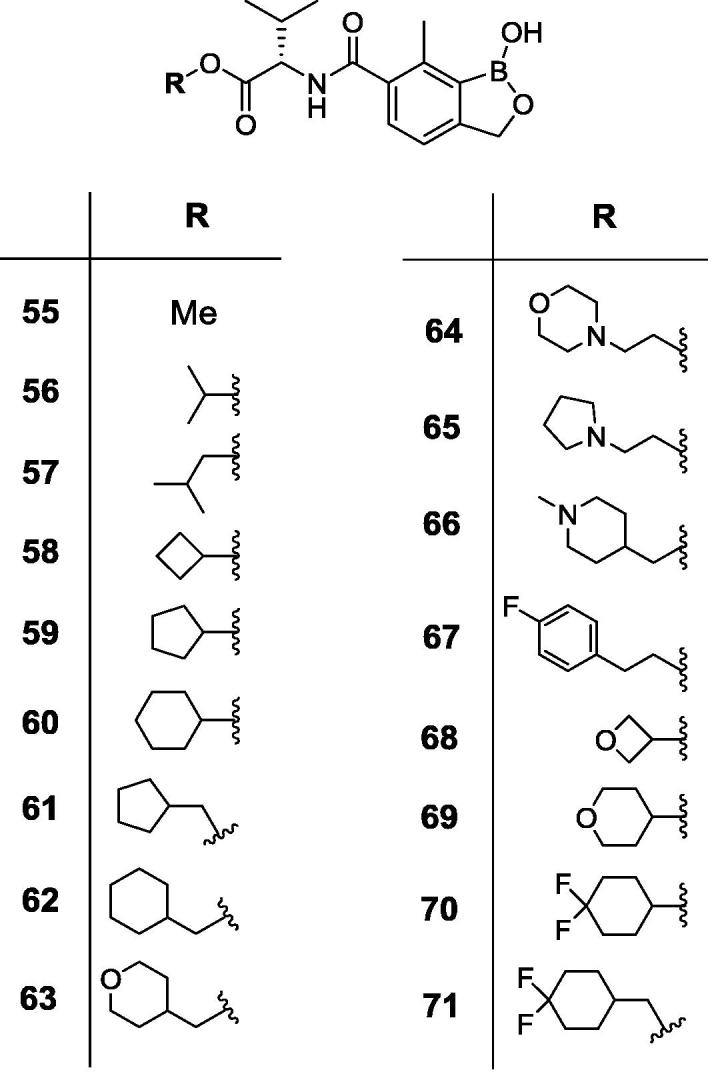
Structures of benzoxaboroles with variation of the left side aliphatic ester groups (**55**–**71**) as compared to analog **5**.

Compounds **1**–**71** were convergently synthesized from three building blocks: the left side alcohols (**72**), amino acid linkers (**73**) and benzoxaborole 6-carboxylic acids (**76**).^3^, ^4^ The general synthetic route is shown in Scheme 1. Reaction of alcohols **72** with *N*-Boc protected amino acids **73** gave ester intermediates **74**, which were treated with dry hydrogen chloride to generate ester amine salts **75**. Condensation of these amine salts **75** with benzoxaborole 6-carboxylic acids **76** provided the final compounds **1**–**71**.

**Scheme 1:**
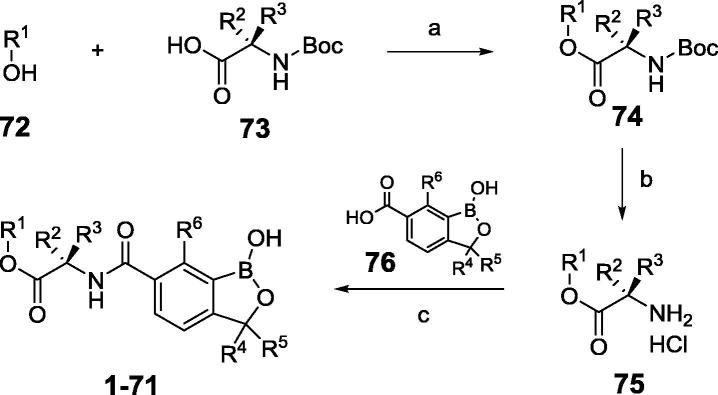
General route for syntheses of **1**–**71**. Reagents and conditions: (a) DCC, DMAP, DCM, 0–20 °C, 16 h; (b) HCl/EtOAc, 20 °C, 5 h; (c) HOBt, EDCI, TEA, DCM, 0–20 °C, 15 h.

Scheme 2 illustrates the synthesis of 1-hydroxy-7-methyl-1,3-dihydrobenzo[*c*][1,2]oxaborole-6-carboxylic acid (**83**) as an example of key boron intermediates. Esterification of the acid **77** produced the ester **78**, which was formylated to yield **79**. Treatment of **79** with trifluoromethyl sulfonyl anhydride afforded the triflate compound **80**, which was converted to the pinacol boron intermediate **81**. Reduction of **81** and subsequent cyclization under aqueous acidic conditions generated the benzoxaborole ester **82**. Hydrolysis of the ester group in **82** afforded the acid **83**. The experimental procedures for the synthesis of **5** are described in the reference and note section.^5^

**Scheme 2:**
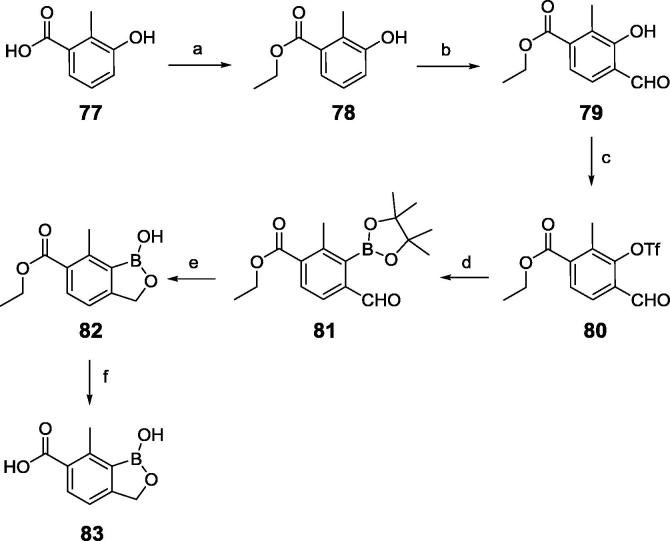
Synthetic route for preparation of **83**. Reagents and conditions: (a) H_2_SO_4_, EtOH, reflux, 24 h; (b) MgCl_2_, (CH_2_O)_n_, TEA, THF, reflux, 14 h; (c) Tf_2_O, pyridine, DMAP, DCM, 0–15 °C, 1 h; (d) Pin_2_B_2_, KOAc, Pd(dppf)Cl_2_, 1,4-dioxane, N_2_, 85 °C, 15 h; (e) NaBH_4_, MeOH, THF, 0–15 °C, 1 h, then HCl, H_2_O; (f) NaOH,H_2_O, 40 °C, 3 h, then HCl for acidification.

Activity of compounds **1**–**71** against *T. congolense* and *T. vivax* was determined using the whole cell assays as described ^6^ and their IC_50_ values are summarized in Table 1.

**Table 1:** Activity of compounds **1**–**71** against *T. congolense* (*T. c.*) and *T. vivax* (*T. v.*).^a^

Compound	IC_50_ (nM)		Compound	IC_50_ (nM)	
	*T. c.*	*T. v.*		*T. c.*	*T. v.*
**1**	4.9	69	**37**	0.78	0.50
**2**	2.0	2.0	**38**	0.68	0.31
**3**	2580	9190	**39**	0.57	0.24
**4**	0.46	0.79	**40**	0.27	0.50
**5**	0.14	1.3	**41**	0.78	0.11
**6**	0.47	2.9	**42**	0.20	0.19
**7**	0.59	0.10	**43**	0.062	NT^b^
**8**	0.28	0.07	**44**	5000	980
**9**	0.18	0.10	**45**	0.37	0.33
**10**	0.22	2.7	**46**	0.42	0.24
**11**	0.16	24	**47**	0.20	0.081
**12**	0.31	19	**48**	<0.005	0.71
**13**	0.23	0.04	**49**	0.39	68
**14**	0.10	0.06	**50**	0.32	0.48
**15**	0.15	0.05	**51**	0.25	0.21
**16**	0.08	0.07	**52**	1.3	0.29
**17**	0.21	0.04	**53**	0.51	0.35
**18**	0.20	0.06	**54**	1.0	0.37
**19**	0.61	0.44	**55**	4.2	3.3
**20**	3.0	1.0	**56**	5.2	14
**21**	28	NT^b^	**57**	0.46	0.26
**22**	0.67	0.92	**58**	0.66	0.69
**23**	37	51	**59**	0.70	0.52
**24**	0.11	0.05	**60**	<0.005	0.78
**25**	3.0	4.7	**61**	0.36	0.21
**26**	2.3	0.05	**62**	0.38	0.16
**27**	3.9	0.71	**63**	0.39	0.09
**28**	0.12	0.38	**64**	2.3	0.78
**29**	0.26	0.10	**65**	9.4	1.2
**30**	0.09	0.25	**66**	5.9	4.1
**31**	0.13	18	**67**	0.43	0.79
**32**	0.45	2.5	**68**	1.8	37
**33**	0.28	26	**69**	0.47	1.6
**34**	0.14	0.07	**70**	0.34	2.28
**35**	0.15	0.09	**71**	0.2	0.14
**36**	0.26	0.14			

a Experimental procedures are described in the reference and note section.^6^

b NT = Not tested.

Lead compound **2** exhibited an IC_50_ of 2 nM against both *T. congolense* and *T. vivax*. The 3,3-dimethyl analog **3** was essentially inactive (IC_50_ = 2580 nM against *T. c.* and 9190 nM against *T. v.*) but better activity was observed for the 7-methyl analog **4** (IC_50_ = 0.46 nM against *T. c.* and 0.79 nM against *T. v.*). We focused future SAR development on compounds incorporating the 7-methyl group, as *in vivo* activity of **4** was superior to that observed for **2** *(vide infra)*. A wide range of substituents, such as halogens, trifluoromethyl, trifluoromethoxy, cyano and methylsulfonyl (**5**–**19** in Fig. 3) on the benzyl ring were introduced to examine their effects on the antiparasitic activity. The majority of these fifteen compounds, with exception of **11** and **12**, were very potent showing IC_50_ values around 1 nM (see Table 1). We next explored variation of the substituent at the 7-position of benzoxaborole (**20**–**27** in Fig. 4). The difluoromethyl (**22**) and ethyl (**24**) analogs had similar activity to that of **5,** but the electron-withdrawing fluoro (**21**) and trifluoromethyl (**23**) analogs were of significantly reduced potency. The amino acid linker was also modified (Fig. 5), with the cyclopropyl (**28**), 2-hydroxyisopropyl (**29**), 2-fluoroisopropyl (**30**) and spirocyclobutyl (**32**) analogs exhibiting potency similar to **5**, but the spirocyclopropyl analog (**31**) exhibited decreased activity against *T. v.* parasite. Replacement of the 4-fluorophenyl in **5** with various heteroaryl groups (**33**–**48**, Fig. 6) resulted in the excellent activity in all cases except the NH-imidazole analog **44**. Introduction of basic nitrogen-containing groups on the benzyl ester (**49**–**54**, Fig. 7) provided compounds **50**–**54** that were generally similar to **5**. Lastly, aliphatic and heterocyclic esters (**55**–**71**, Fig. 8) were synthesized and many of these had IC_50_ values less than 1 nM as shown in Table 1.

Selected compounds were screened in both mouse and bovine *in vitro* metabolic stability assays (mouse S9 and bovine S9), as summarized in Table 2. These two species were chosen because the primary *in vivo* assays were conducted in mice, and the target animal of this research program is cattle. As shown in Table 2, out of 36 compounds tested, 27 compounds had Cl_int_ < 10 µL/min/mg protein in both mouse and bovine S9 assays suggesting moderate to excellent *in vitro* metabolic stability. We evaluated the efficacy of selected compounds in two *in vivo* mouse models of infection, against *T. congolense* and *T. vivax*, respectively. Mice were infected with either 1 × 10^5^ *T. c.* parasites or 1 × 10^4^ *T. v.* parasites, and then treated with a test compound via intraperitoneal administration for 1, 2 or 4 consecutive days. The mice were then monitored for the presence of parasitemia for up to 60 days post treatment.^7^ We tested in *T. c.* model first, then followed up with *T. v.* for interesting compounds. As shown in Table 2, the 7-methyl analog **4** was superior to the 7-unsubstituted analog **2** in both *T. c.* and *T. v.* mouse models of infection. Of the 38 compounds tested with the *in vivo* mouse models, seven compounds (**5**, **8**, **33**, **34**, **49**, **62** and **71**) demonstrated ≥50% curative efficacy in the *T. c*.-infected mouse model and 100% curative efficacy in the *T. v*.-infected mouse model, when tested as a single dose of 10 mg/kg. To select further from these seven compounds, four (**5**, **8**, **33** and **71**) had ≥75% curative efficacy in the *T. c*.-infected mouse model at a single dose of 10 mg/kg, and two (**5** and **8**) showed 100% curative efficacy. These two compounds were further tested at a single 5 mg/kg dose, but were unable to cure the *T. c.*-infected mice. We selected compound **5** (**AN11736**) to progress to exploratory studies to determine the efficacy and safety in a preliminary formulation against induced infections of *T. vivax* and *T. congolense* in cattle.^8^ **AN11736** demonstrated 100% curative efficacy with a single intramuscular injection of 10 mg/kg against both *T. congolense* and *T. vivax* in cattle.

**Table 2:** *In vitro* metabolic stability and *in vivo* mouse efficacy of selected compounds.^a^

Compound	Cl_int_ (µL/min/mg protein)		Efficacy in mouse model^b^	
	Mouse S9	Bovine S9	*T. c.*	*T. v.*
**2**	NT^c^	NT^c^	0/5 (2 × 10)	5/5 (4 × 10) 1/5 (1 × 10)
**4**	NT^c^	NT^c^	4/5 (2 × 10)	5/5 (2 × 10) 4/4 (1 × 10)
**5**	5.4	9.3	5/5 (2 × 10) 4/4 (1 × 10) 0/4 (1 × 5)	5/5 (2 × 10) 5/5 (1 × 10) 4/4 (1 × 10)
**6**	17	9.1	0/4 (1 × 10)	NT^c^
**8**	7.9	5.6	4/4 (1 × 10) 0/4 (1 × 5)	4/4 (1 × 10)
**14**	9.1	2.7	0/4 (1 × 10)	NT^c^
**16**	15	8.9	4/4 (2 × 10) 1/4 (1 × 10)	NT^c^
**17**	14	10	0/4 (1 × 10)	NT^c^
**18**	9.7	3.1	1/4 (1 × 10)	NT^c^
**19**	2.6	1.3	0/4 (1 × 10)	NT^c^
**20**	1.5	1.3	0/5 (4 × 10)	NT^c^
**21**	<1	<1	1/4 (2 × 10)	4/4 (2 × 10)
**22**	1.4	0.9	0/4 (1 × 10)	NT^c^
**24**	21	23	1/4 (2 × 10)	NT^c^
**26**	32	32	0/4 (1 × 10)	NT^c^
**29**	<1	<1	0/4 (1 × 10)	NT^c^
**30**	3.7	3.1	0/4 (1 × 10)	NT^c^
**31**	<1	<1	0/4 (1 × 10)	NT^c^
**33**	6.3	2.0	3/4 (1 × 10)	4/4 (1 × 10)
**34**	11.8	4.8	2/4 (1 × 10)	4/4 (1 × 10)
**35**	5.2	7.8	0/4 (1 × 10)	NT^c^
**39**	1.6	3.9	0/4 (1 × 10)	NT^c^
**41**	1.3	1.8	0/4 (1 × 10)	NT^c^
**46**	7.4	7.0	1/4 (1 × 10)	NT^c^
**47**	4.8	10	0/4 (1 × 10)	NT^c^
**48**	4.0	23	1/4 (1 × 10)	NT^c^
**49**	1.5	4.0	2/4 (1 × 10)	4/4 (1 × 10)
**50**	8.6	12.7	1/4 (1 × 10)	4/4 (1 × 10)
**51**	1.8	5.0	0/4 (1 × 10)	4/4 (1 × 10)
**52**	1.8	6.0	0/4 (1 × 10)	NT^c^
**53**	5.4	6.8	0/4 (1 × 10)	NT^c^
**54**	5.5	3.8	0/4 (1 × 10)	NT^c^
**55**	<1	3.1	0/4 (1 × 10)	NT^c^
**62**	10	20	2/4 (1 × 10)	4/4 (1 × 10)
**63**	0.9	1.5	1/4 (1 × 10)	4/4 (1 × 10)
**69**	0.7	0.6	1/4 (1 × 10)	1/4 (1 × 10)
**70**	1.5	0.3	1/4 (1 × 10)	4/4 (1 × 10)
**71**	6.4	8.1	3/4 (1 × 10)	4/4 (1 × 10)

a Methods for testing compound efficacy in mouse models are described in the reference and note section.^7^

b The efficacy data in the table is presented as n/m (q × 10), where n = number of mice survived, m = total number of mice in the study group, q = how many time dosed, and 10 = 10 mg/kg.

c NT = Not tested.

In summary, a novel series of *l*-valinate amide benzoxaboroles was discovered to be active against *T. congolense* and *T. vivax*, which are the main causative agents of Animal African Trypanosomiasis (AAT) in cattle. Two compounds (**5** and **8**) showed 100% curative efficacy in both *T. c*.- and *T. v.*-infected mice with a single dose of 10 mg/kg. Compound **5** (**AN11736**) demonstrated 100% curative efficacy with a single IM dose of 10 mg/kg against both *T. congolense* and *T. vivax* in cattle for a duration of 100 days. **AN11736**, as a novel chemical entity, was selected as a potential developmental candidate for the treatment of AAT.
